# Long-Term Evaluation of Women Referred to a Breast Cancer Family History Clinic (Manchester UK 1987–2020)

**DOI:** 10.3390/cancers12123697

**Published:** 2020-12-09

**Authors:** Anthony Howell, Ashu Gandhi, Sacha Howell, Mary Wilson, Anthony Maxwell, Susan Astley, Michelle Harvie, Mary Pegington, Lester Barr, Andrew Baildam, Elaine Harkness, Penelope Hopwood, Julie Wisely, Andrea Wilding, Rosemary Greenhalgh, Jenny Affen, Andrew Maurice, Sally Cole, Julia Wiseman, Fiona Lalloo, David P. French, D. Gareth Evans

**Affiliations:** 1 Nightingale Breast Screening Centre & Prevent Breast Cancer Unit Wythenshawe Hospital, Manchester University NHS Foundation Trust, Manchester M23 9LT, UK; ashu.gandhi@mft.nhs.uk (A.G.); sacha.howell@manchester.ac.uk (S.H.); mary.wilson@mft.nhs.uk (M.W.); Sue.astley@manchester.ac.uk (S.A.); michelle.harvie@manchester.ac.uk (M.H.); Mary.pegington@manchester.ac.uk (M.P.); lester.barr@mft.nhs.uk (L.B.); aetab2@btinternet.com (A.B.); elaine.harkness@manchester.ac.uk (E.H.); penny.hopwood@icr.ac.uk (P.H.); julie.wisely@mft.nhs.uk (J.W.); Andrea.Wilding@MFT.NHS.UK (A.W.); rosemary.greenhalgh@mft.nhs.uk (R.G.); jenny.affen@mft.nhs.uk (J.A.); Andrew.maurice@nhs.net (A.M.); Sally.cole@mft.nhs.uk (S.C.); julia.wiseman@mft.nhs.uk (J.W.); fiona.lalloo@mft.nhs.uk (F.L.); Gareth.Evans@mft.nhs.uk (D.G.E.); 2 Manchester Breast Centre, The Christie Hospital, Manchester M23 9LT, UK; 3 Division of Cancer Sciences, Medicine and Health, University of Manchester, Manchester Academic Health Science Centre, Manchester M23 9LT, UK; 4 Division of Informatics, Imaging & Data Sciences, School of Health Sciences, Faculty of Biology, Medicine and Health, University of Manchester, Manchester M13 9PT, UK; 5 Department of Surgery, King Edward VII’s Hospital, London W1G6AA, UK; 6 Clinical Trials and Statistics Unit, The Institute of Cancer Research, London SM25PT, UK; 7 Department of Psychology, University Hospital of South Manchester NHS Trust, Wythenshawe, Manchester M23 9LT, UK; 8 Manchester Centre for Genomic Medicine, Manchester University Hospitals NHS Foundation Trust, Manchester M23 9LT, UK; 9 Division of Psychology and Mental Health, School of Health Sciences, Manchester Centre of Health Psychology, University of Manchester, Manchester M23 9LT, UK; david.french@manchester.ac.uk; 10 NW Genomic Laboratory Hub, Manchester Centre for Genomic Medicine, Manchester University Hospitals NHS Foundation Trust, Manchester M13 9WL, UK; 11 Faculty of Biology, Division of Evolution and Genomic Sciences, School of Biological Sciences, Medicine and Health, University of Manchester, Manchester Academic Health Science Centre, Manchester M23 9LT, UK

**Keywords:** family history, breast cancer, risk, genes, screening, prevention

## Abstract

**Simple Summary:**

This study reports the management of women at high risk for breast cancer over a 33 years period. The aim was to summarize the numbers seen and to report the results of our studies on gene testing, the outcomes of screening and the success of preventive methods including lifestyle change, chemoprevention and risk-reducing mastectomy. We also discuss how the clinical Family History Service may be improved in the future.

**Abstract:**

Clinics for women concerned about their family history of breast cancer are widely established. A Family History Clinic was set-up in Manchester, UK, in 1987 in a Breast Unit serving a population of 1.8 million. In this review, we report the outcome of risk assessment, screening and prevention strategies in the clinic and propose future approaches. Between 1987–2020, 14,311 women were referred, of whom 6.4% were from known gene families, 38.2% were at high risk (≥30% lifetime risk), 37.7% at moderate risk (17–29%), and 17.7% at an average/population risk who were discharged. A total of 4168 (29.1%) women were eligible for genetic testing and 736 carried pathogenic variants, predominantly in *BRCA1* and *BRCA2* but also other genes (5.1% of direct referrals). All women at high or moderate risk were offered annual mammographic screening between ages 30 and 40 years old: 646 cancers were detected in women at high and moderate risk (5.5%) with a detection rate of 5 per 1000 screens. Incident breast cancers were largely of good prognosis and resulted in a predicted survival advantage. All high/moderate-risk women were offered lifestyle prevention advice and 14–27% entered various lifestyle studies. From 1992–2003, women were offered entry into IBIS-I (tamoxifen) and IBIS-II (anastrozole) trials (12.5% of invitees joined). The NICE guidelines ratified the use of tamoxifen and raloxifene (2013) and subsequently anastrozole (2017) for prevention; 10.8% women took up the offer of such treatment between 2013–2020. Since 1994, 7164 eligible women at ≥25% lifetime risk of breast cancer were offered a discussion of risk-reducing breast surgery and 451 (6.2%) had surgery. New approaches in all aspects of the service are needed to build on these results.

## 1. Introduction

In the 1980s, the rising incidence of breast cancer (BC) and the introduction in the UK of the NHS National Health Service Breast Screening Programme (NHSBSP) led women with a family history of the disease to seek advice concerning management of their personal risk. In response to concerns expressed by primary care physicians and colleagues within our breast oncology service, we established a referral Family History Clinic (FHC) in Manchester, UK, in 1987 with a cancer genetics service (CGS) initiated in 1990 (DGRE, FL). The clinic serves a catchment population of 1.8 million, (just over half the population of Greater Manchester), although women at high-risk may be specifically referred to the centre from a population of approximately 5 million in North West England.

The aims of the FHC were to introduce a service for the estimation and management of BC risk for women with familial risk and to evaluate the short- and long-term effectiveness of the clinic. At presentation, an individual’s risk was explained, annual breast screening initiated and advice given concerning diet and lifestyle factors which might affect risk. Later, chemoprevention (1992 as part of the IBIS I clinical trial), genetic testing (1994) and risk-reducing surgery (1994) were introduced. In 1994, we published local guidelines for the management of women with a family history of BC [1]. These were followed by national guidelines for the UK [2,3] and the USA [4,5].

Management was undertaken by a multi-disciplinary team. Following referral, each woman was sent a questionnaire to assess family history and breast factors and, if eligible, offered a clinic appointment. Women were initially seen by geneticists (DGRE, FL) or medical oncologists (AH, SJH). Breast examination was undertaken and advice given by specially trained nurses (RG, JW, AW) and annual mammography and MRIs performed by radiologists within the Breast Unit (represented by MW and AM). Risk-reducing surgery was performed by a team of surgeons (represented by AB, AG and LB). Quality of life aspects of risk communication, mutation testing and risk-reducing mastectomy (RRM) were an integrated part of the FHC clinical and research agenda provided by psycho-oncologists and a health psychologist (PH, JW, DF).

The aims of this paper were to present the results of each aspect of the service and to suggest potential future improvements. The results include the numbers of referrals, estimation of their BC risks and results of genetic testing, screening and uptake to lifestyle prevention, chemoprevention and risk-reducing surgery interventions. The main quality of life outcomes are also reported. The second half of this paper then suggests improvements to the service based on our own studies and those of others.

## 2. Results

### 2.1. Referrals to the Clinic

**Figure 1:**
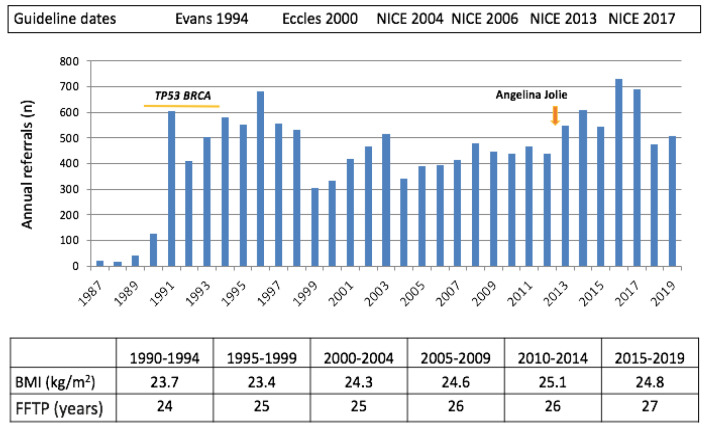
Annual referrals to the Manchester FHC between 1987 and 2020. Increases in referral were seen during the period when the first breast cancer genes, *TP53* and *BRCA1/2*, were identified and also related to the publicity surrounding Angelina Jolie when she indicated that she was a *BRCA1* PV carrier. The increase in median body mass index (BMI) and median age at first full-term pregnancy (FFTP) over this period are shown. Over the period of 33 years, lifetime risk of BC in the population increased from 1 in 12 to 1 in 8, an increase presumed to be associated with change in modifiable risk factors. These trends were apparent in the FHC. For example, the median age of first full-term pregnancy increased from 24 years to 27 years (*p* < 0.001), and median BMI at clinic entry increased from 23.7 to 24.8 kg/m^2^ (*p* < 0.001).

Over the period from September 1987 to September 2020, 14,311 women were referred to the clinic. Referrals were from primary care (GPs, 55.9%), from secondary care (mainly breast surgeons, 15.1%) and from the local Clinical Genetics Service for further follow up after risk assessment (20.9%). Women were also referred from a research study (notably, 3.1% from PROCAS—Prediction Risk Of Cancer At Screening, [6]) or from “other” sources such as relatives at risk attending with a proband (5.0%). The age at entry ranged from 16–81 years (median 39.9; interquartile range (IQR) 33.9–46.9, with 83% of women (11,878/14,311) below age 50 (Table 1). The number of referrals by year is shown in Figure 1 and ranged between approximately 300–700 per year following the initial 3 years lead in the period after the clinic was established.

### 2.2. Estimated Lifetime BC Risk

Risk was estimated initially by a modification of the Claus method with the addition of hormonal and lifestyle factors, such as age of first pregnancy and BMI, and by the Tyrer–Cuzick and BOADICEA models from 2004 [7,8,9]. We demonstrated that the modified Claus and the Tyrer–Cuzick models gave similar distributions of risk accurately, but the Gail model underpredicted risk [10,11].

Risk was reported as moderate (17–29% lifetime risk) and high (30%+) according to our original clinic guidelines (Evans 1994) and thereafter using the NICE Guidelines risk categories (2004) [1,3]. Overall, 44.6% of women were at high-risk (including 6.4% either with a pathogenic variant (PV) or a known PV in a close family member), 37.7% at moderate risk and 17.7% at average risk (Table 1). Following assessment of the referral questionnaire, women at average risk were referred back to primary care. It was clear that more higher risk women were referred to the Clinical Genetics Service reflecting a referral pathway from primary care to the Clinical Genetics Service for the highest risk women. (Table 1). After excluding referrals at average risk, 43.7% (*n* = 3397) of women directly referred to the clinic from primary and secondary care were estimated to be at high-risk and 56.7% (*n* = 4373) were at moderate risk.

### 2.3. Risk Perception and Cancer Worry

Risk was uniformly given as a proportion (e.g., 1 in 4 or 1 in 5). Our early studies reported that women had a relatively inaccurate perception of personal and population risk at presentation which improved when reassessed after risk counselling [12,13]. In another study, we demonstrated that the proportion of women with accurate personal risk perceptions significantly improved after risk counselling from 12% pre-counselling to 67% 3 months post-counselling (*p* < 0.001), which was maintained for 1 year [14]. Reassuringly, this improvement in accuracy of women’s risk appraisal was not associated with increased anxiety. A subsequent analysis of questionnaire data from 500 FHC attendees over time indicated that BC risk counselling reduced self-reported cancer worry in women who initially overestimated their risk, with no significant change in levels for other risk perception groups, even if the risk was greater than they had estimated pre-counselling [15,16].

### 2.4. Genetic Testing

Genetic testing in the clinic began in 1991 just after the discovery of *TP53* and then the *BRCA1* (1994) and *BRCA2* (1995) genes [17,18,19]. Initially, testing was by single-strand confirmation polymorphism (SSCP) and protein truncation testing (PTT) [20], then, from 2001, Sanger sequencing all coding exons [20], and from 2013, next generation sequencing [21]. All samples, including retrospectively, were tested for large deletions and duplications by multiple ligation-dependent probe amplification (MLPA). All mutations detected by PTT or SSCP were confirmed by sequencing. From 2004, the probability of a *BRCA1* or *BRCA2* PV in the family was estimated using the Tyrer–Cuzick model [8] the BOADICEA model [9] or the Manchester Score [20,21]. The NICE Guidelines (2004) [3] initially indicated that the proband required the probability of a PV in BRCA1/2 of ≥20% for genetic testing in England and Wales [3]. In 2013, this was reduced to a ≥10% likelihood [22]. At present, gene testing in the UK is restricted to the *BRCA1, BRCA2,* and *PALB2* genes (National Genomic Test Directory (2020)) [23].

Of the 14,311 referrals to the FHC, *BRCA1* and *BRCA2* testing was completed for 4168 individuals (29.1%) or their affected family member. A total of 736 women (5.1% of the whole FHC cohort and 17.6% of those tested) were identified as *BRCA* PV carriers (*BRCA1* = 364, *BRCA2* = 372). However, only 2.5% of unaffected direct referrals to the FHC without a known gene in the family subsequently tested positive. No systematic approach to testing for PVs in other genes was undertaken. However, 35 potential BC and other risk genes were tested on a research basis in a subpopulation of 808 women unaffected by breast cancer representative of the risk distribution of direct referrals to the FHC [24]. Of the 808 tests there were 29 (3.6%) with PVs in other potentially actionable genes (*ATM* = 11, *CHEK2* = 11, *PALB2* = 7). These data indicate that approximately 6.1% of women directly referred to the FHC carry a PV in one of the actionable BC-associated genes.

### 2.5. Mammographic Screening

Annual mammography and clinical breast examination were offered from the inception of the clinic. Women were screened between the ages of 35 and 50 years (moderate risk) or 35 to 60 years (high risk), plus from 5 years younger than the earliest family diagnosis of BC. These screening periods were in accordance with an initial in-house protocol [25] and later, NICE guidelines [3,22]. Women with *BRCA1* and *BRCA2* PVs and others of equivalent high risk were screened by annual MRI between age 30 and 50 and annual mammography from age 30 to 70 according to NICE Guidelines [3,22].

Between 1987 and 2020, there were 129,119 women years of follow up; 646 BCs occurred prospectively, giving an annual incidence rate of 5.0 per 1000 which was approximately 1.7 times higher than the general population’s annual rate of 3 per 1000 women aged 50–75 in the NHSBSP. Three hundred and ninety-four breast cancers occurred whilst on the screening programme or within 18 months of a screen. The majority of invasive cancers were lymph node negative (72.9%), small (≤20 mm, 73.2%) and stage 1 (61.4%). Cancers in women with *BRCA1* and *BRCA2* PVs were smaller overall with 75.0% and 85.4% being ≤20 mm, respectively, reflecting the benefits of MRI screening [26]. Breast cancer-specific survival at 10 years was 91.3% (87.4–94.0), compared with the current 10 year survival from BC in England, from 2013–2017, of 75.9% (74.9–77.0). Overall, 30.5% (92/322) of invasive cancers were oestrogen receptor negative (ER–) and 11/51 (21.5%) of carcinoma in situ (not assessed 21) were ER–.

### 2.6. Lifestyle Prevention

Excess weight, weight gain, sedentary lifestyle and high alcohol intake were established risk factors for BC before initiation to the FHC, and so verbal and standard written lifestyle advice was given to all women referred to the clinic. Lifestyle risk factors were common amongst high-risk women in the FHC with a similar prevalence of unhealthy lifestyles to women in the general population (57% are overweight/obese, 30% report <150 min moderate intensity physical activity/week and 45% have alcohol intakes of >14 units per week) [27]. Adult weight gain is a well-documented risk factor for BC (6% increased risk per 5 kg gain) [28]. We and others have reported that maintained weight loss of 5% or more before or after menopause in an unselected population cohort is associated with reductions of 39% and 23%, respectively, in the risk of post-menopausal BC [29]. Thus, women were advised to avoid weight gain if at a healthy weight and reduce weight by at least 5% if overweight/obese. Current guidelines include advising at least 150 min of moderate exercise per week and no more than 14 units of alcohol per week.

Since 2001 we conducted a series of randomised studies to determine the optimal methods for introducing and supporting weight control and other lifestyle changes amongst women in the FHC. These indicated that intermittent energy restriction (2 days of 50–60% energy restriction and 5 days of normal healthy eating/week) was associated with greater reductions in weight and insulin resistance than continuous energy restriction [30,31]. We also demonstrated that remotely supported (i.e., web and phone) weight loss/lifestyle behaviour change programmes are feasible and particularly effective amongst higher risk women producing ≥5% weight loss in approximately 60% of women at 12 months [32]. Uptake to these weight loss trials amongst moderate and high-risk women was between 14% and 27%.

### 2.7. Chemoprevention

Chemoprevention was not available at the inception of the FHC. However, women were randomised to tamoxifen or placebo in the IBIS-I trial (ISRCTN91879928). Between 1992 and 2001 and to anastrozole or placebo in the IBIS-II trial (SRCTN31488319). Between 2003 and 2012 [33,34]. Of the 7865 women invited to join these trials, 1003 women (12.8%) agreed (Table 2). These and other studies resulted in tamoxifen use being advised by NICE in the 2013 guidelines and anastrozole in the 2017 guidelines [22]. From 2013, 5121 women were invited to take either drug as part of management. To date, 282 have chosen chemoprevention and a further 284 in clinical trials so that overall 10.8% of eligible women have accepted treatment since 2013 (Table 2).

### 2.8. Risk-Reducing Mastectomy

Risk-reducing mastectomies were being performed, particularly in the USA, at the time of inception of the clinic. In 1994, we decided to offer this service in the FHC. Since then, 7164 women with a lifetime BC risk ≥25%, including *BRCA1/2* PV carriers, were offered a discussion concerning RRM according to a published protocol [35]. Of a total cohort of 7195 women at a ≥25% lifetime risk of BC, 451 (6.2%) without a current or previous breast cancer diagnosis elected to undergo RRM. Uptake of RRM was 49.3% in 479 *BRCA1/2* PV carriers and 5.2% in 6685 in non-carriers (9.4% for 1261 women at a ≥40% lifetime risk (non-BRCA), 4.9% in 3561 women at 30–39% risk and 3.0% in 1783 women at 25–29% lifetime risk).

In Cox regression analyses, factors which independently predicted risk-reducing mastectomy uptake included either the death of a sister with BC <50 years or mother <60 years, having children, having a breast biopsy or younger age at assessment (<30 years).

Of the 451 women who underwent RRM, four developed post-surgery BCs (all in *BRCA1/2* PV carriers) compared to 94 expected over a period of follow up of 7894 years, giving a risk reduction of 95.8%.

Twenty women (5.7%) had no reconstruction, whereas 352 (78%) had implant-based reconstruction (nipple sparing in 31% of these) and 63 (14%) flap-based reconstruction. The number of planned surgical procedures per patient was 2.41 ± 1.11 SD [36].

Two studies assessing psychological distress in our FHC patients undergoing risk-reducing mastectomy have been published [37,38]. Between 1995 and 1999, quality of life was assessed in 52 of 76 (79%) women undergoing surgery one-year post-operatively. At this point, 1 in 6 women had high scores for mental health problems on the General Health Questionnaire but for most, psychological distress appeared to be comparable with women at high risk who did not have surgery. Body image changes on the Body Image Scale were generally minor in degree with the most frequently reported changes reported in sexual attractiveness (55% responders), feeling less physically attractive (53%) and self-consciousness about appearance (53%). For the majority of women there was no evidence of significant mental health or body image problems in the first 3 years following RRM. Careful pre-operative preparation and long-term monitoring was advocated. In a second study, 79 women who chose to have surgery were compared with 64 women who declined surgery [38]. The main findings were that risk-reducing mastectomy reduced psychological morbidity and anxiety and did not have a significant detrimental impact on women’s body image or sexual functioning.

## 3. Discussion

We summarised the developments that occurred since the inception of the clinic including updated models for risk and gene testing estimation (i.e., Tyrer-Cuzick v8 2017 [39], BOADICEA-V2019 [40], the Manchester Score [20,21] consistent use of mammography and MRI (NICE 2013 [22], a more po-active approach to lifestyle change [32], chemoprevention [41] and RRM [42]. We now suggest potential improvements to the service in each of the areas considered above based on our own studies and those of others.

### 3.1. Referrals

The numbers of referrals have remained relatively stable over the years and are still mainly instituted by women concerned about their family history and primary and secondary care clinicians who refer according the NICE familial breast FHC guidelines [3]. Our own and the reports of others indicate that approximately 10% of women in the UK have a first degree relative with BC; however, we estimate that <20% of these are referred [43]. It is interesting that referrals to FHCs and Clinical Genetics Services increased over two-fold after Angelina Jolie made her *BRCA1* PV carrier status and breast and ovarian surgery public [44,45]. This suggests that lack of awareness of the services may be an issue. Lack of uptake may also be related to a complex referral system which requires a visit to primary care for referral and the completion of extensive questionnaires. The latter is illustrated in our study of risk estimation in women undergoing breast screening. Thirty-seven percent of women invited completed a two-page questionnaire concerning their risk factors [6]. The cohort included 13% of women with a first-degree relative with breast cancer. Of 673 women found to be at high risk and invited for counselling and treatment at the FHC, 500 (74.3%) attended [46]. These data indicate a greater interest in risk management if the system is streamlined, suggesting that progress may best be made by more effort to align risk estimation with screening programmes. Referral would then be less dependent on health care professionals and would make it a more routine and streamlined service.

### 3.2. Risk Estimation

We demonstrated that the modified Claus model used in the clinic before 2004 gave similar results to the Tyrer–Cuzick model, suggesting consistent risk estimation for the duration of the clinic to date [10,11]. However, several studies indicate that the accuracy of risk estimation increases with the number of risk factors that are incorporated into the models used [47,48,49,50]. Recently, mammographic density (MD) and polygenic risk scores (PRS) based on single nucleotide polymorphism (SNP) results) have been added to risk models such as Tyrer–Cuzick (v8) and BOADICEA V [39,40]. In patients under follow up at the FHC, we assessed the effect on risk of incorporating the first 18 BC risk-associated SNPs discovered into the Tyrer–Cuzick model [51,52]. Adding SNP18 resulted in a change to the original given risk using Tyrer–Cuzick (version 6) in half the population of women: 25% had an increase in risk and 27% had a decrease in risk, indicating the potential importance of additional risk factors [53].

In the screening population we demonstrated that when both mammographic density and a PRS score were added to the Tyrer–Cuzick (v8), the proportion of women at elevated risk (>5% 10 years risk) increased from 12% to 18%. Ten percent of women changed from average to high risk and 4% from high to average [54,55]. These studies illustrate that using standard models may give erroneous risks and adding more risk factors may result in more appropriate management. However, more work is required to routinely apply optimal risk models in the clinic and deal with change in risk estimation over time.

### 3.3. Genes

Of the women directly referred to the FHC in which there was no currently known PVs, 2.5% were *BRCA1/2* PV carriers and 3.6% were found to have PVs in other genes after multigene panel testing [24]. This low pickup rate reflects that over 50% of referrals were at moderate risk and many of those at higher risk had undergone testing in themselves or their family (Table 1). At the FHC, much time was used to calculate the probability that the proband or her family are likely to carry a breast cancer gene PV. At present our Clinical Genetics Service forwards primary care referrals for women unlikely to carry a PV immediately to the FHC; conversely, we refer relatives of known PV carriers to the Clinical Genetics Service. A simple method where primary and secondary care physicians could estimate PV risk and refer appropriately would be invaluable. A more widespread use of the simple Manchester Score needs to be evaluated in this regard [20,21]. Currently, the NHS guidelines to the UK genetics departments allow estimation of *BRCA1/2* and *PALB2* (as well as syndromic genes where indicated such as *PTEN* in Cowden disease) [23]. The recent report illustrating the nine genes (*BRCA1, BRCA2*, *PALB2, CHEK2, ATM, CDH1, STK11, PTEN, TP53)* in which PVs more than doubles the risk of BC and three genes at the two-fold threshold (*RAD51C, RAD51D, BARD1)* might allow better selection for appropriate gene testing and a reduction in the need for multigene testing which, in the UK, is in the commercial sector [24].

### 3.4. Breast Screening

Mammographic screening in this at-risk population detects more cancers annually (5/1000 screened) than in the national programme (3/1000) as expected. It also results in the detection of smaller, better risk cancers. Three studies in the UK, one in our clinic and two in association with other clinics in the UK indicated that screening at-risk women results in a survival advantage compared (in non-randomised trials) with age matched populations [56,57,58]. The latest study was designed to assess the value of screening both moderate- and high-risk women from age 35–39 and confirmed a survival advantage even in the moderate-risk group [58].

Countries where national screening programmes begin at age 40 will already be screening in the high-risk groups. A review of the value of screening from age 40 in the general population concluded it was of equivocal value [59]. In the UK, screening is every 3 years for all from the age of 50, but a recent study now suggests a survival advantage in the general population when screening begins annually at the age of 40. This may lead to a change in UK policy [60]. The results of two randomised trials of risk adapted screening will inform a potential change in policy since they both screen women from age 40 onwards (WISDOM [61] MyPebs UNICANCER 2018 [62]). Further, a programme of work carried out in Manchester is considering how best to implement risk adapted screening to optimise the ratio of benefits and harms, including how to include ethnically minority women and to minimise harms of screening for women at low risk [63].

In countries where screening begins at 50 (e.g., the UK), consideration should be given to offering all women a one-off mammogram at age 40, together with risk estimation to determine future screening frequency. Mammographic density could be assessed automatically using artificial intelligence methods [64,65] and SNPs used only to determine precise risk where needed. The trials of risk and density-adapted screening and determination of the value of supplemental imaging techniques, such as whole breast ultrasound, contrast-enhanced mammography and abbreviated MRI, would further refine the management advice offered to women with high MD. Currently, at the FHC, women are offered an MRI if they carry a PV of a high-risk gene or if they have a 10 year risk of ≥8% aged 30 or ≥12% aged 40, based on the finding in trials that MRIs detect smaller tumours and may offer a survival advantage [66,67,68,69,70]. However, neither MRIs nor ultrasounds are routinely available (or proven) for a large group of women at increased risk outside those in very high-risk groups.

### 3.5. Lifestyle Advice

Women at high risk who have a high BMI [71], low physical activity levels, high alcohol intake [72] and smoke [73] have proportionately higher BC risks than similar women at population risk [71,72,73]. These potentially modifiable risk factors have also been associated in women at high risk due to the fact of family history or high PRS [72,73]. Thus, there is a rationale for focussing on lifestyle change in this higher risk group, and it is probable that the more avenues to promote change that are pursued, the greater the likelihood of success [74].

Women at high risk present a challenge for achieving lifestyle behaviour change. Firstly, some women can view their BC risk as unchangeable because of their family history [75]. This is consistent with a large body of literature that indicates education around disease risk does not alter behaviour by itself and that people require an appropriate level of support to achieve and sustain lifestyle behavioural change [76]. Lifestyle behaviour change programmes need to address the often complex psychological issues amongst women who have a high burden of cancer diagnoses and bereavements in their family. For many women, the majority of excess weight is acquired between the age of 18 and 35 years [76], indicating that lifestyle programmes should begin at younger ages. Interviews with young, high-risk women (under 35 years) in our FHC indicate that those women require a supportive weight control lifestyle programme that is remotely accessible, provides a point of contact within the high-risk service and promotes general wellbeing as well as cancer risk reduction [76]. There is a need for wider testing of low-cost programmes for lifestyle prevention which can reach and engage the maximum number of women across the network of UK FHCs.

### 3.6. Chemoprevention

The reduction of risk of BC by 30–50% by the use of SERMs and AIs such as tamoxifen, raloxifene and anastrozole are well known [77]. More recently, long-term follow up of the IBIS I and IBIS II trials indicate that risk reduction continues long after the usual five-year prescription period [41,78]. A recent analysis by NICE indicates that the use of anastrozole, in particular, is cost saving to the NHS in women at moderate to high risk of BC. However, whilst reduction of risk is of benefit, none of the trials to date have shown a survival benefit. This has led to the suggestion that premarin should be used for women at least 5 years post-menopausal and without a uterus, since in this group the Women’s Health Initiative trial use was associated with a survival advantage [79].

Our report of recent uptake of chemoprevention being relatively low at 10% is consistent with many but not all studies [80,81]. Part of the reason for the low uptake concerns the side effects, although our own and other studies show that the observed frequency of side effects are comparable to controls [33,34,81,82,83,84]. We found four themes associated with low uptake: the perceived impact of side effects, the impact of others’ experience on beliefs about tamoxifen, tamoxifen as a “cancer drug” and the daily reminder of cancer risk [80]. These reasons are understandable and consistent with other studies. Future developments require better communication of the pros and cons of therapy and alternative approaches including low dose or topical tamoxifen [85]. New agents such as antiprogestins [86], and denosumab [87] are currently being trialled in the FHC and elsewhere.

### 3.7. Risk-Reducing Surgery

Historically, our unit performed approximately 10–12 operations per year. With recent increases in publicity surrounding RRM [44], this has increased approximately three-fold in our own and other units [45]. The seminal paper by Hartmann [88] indicated BC a risk reduction of 92%, similar to our observed reduction of 95.8%. Over the years, our surgical approaches have evolved to reflect refinements in surgical technique and improved technology. Initially, mastectomy inevitably involved sacrifice of the nipple areolar complex, and immediate reconstructions relied exclusively on submuscular implant placement or use of the transverse rectus abdominus flap. In recent years, with the increasing appreciation of patient reported outcome measures in women undergoing risk reducing surgery [89], surgeons have sought more aesthetically focussed reconstruction options whilst not compromising risk-reduction principles. This has allowed the safe introduction of skin sparing and nipple sparing mastectomy [90] and autologous reconstruction with deep inferior epigastric perforator flaps [91] or single-stage prepectoral implant-based reconstruction [92]. The use of acellular dermal matrices has revolutionised implant-based reconstruction, allowing structural support of implants within a reconstruction to mimic natural breast ptosis [93]. Further improvements may come from the use of lipomodelling to improve aesthetics and thus patient satisfaction [94].

In *BRCA* PV carriers, RRM results in an improvement in survival, especially in women with *BRCA1* PVs, a result also found by others [95,96]. There may also be an improvement in women with *BRCA2* PVs with longer follow up [97]. Our previous studies indicated good acceptance and psychological health after RRM [37,38] More recent overviews have emphasised the enormous importance of excellent pre-surgical explanation, the presence of a psychologist in the multidisciplinary team and improved surgical techniques have been emphasised (Braude 2017) [98].

### 3.8. Summary

Here, we summarised the updated results from the Manchester FHC which spans the period from the inception of such clinics in the UK up to the present. A large proportion of these clinics in the UK are associated with Breast Units and work in conjunction with local Clinical Genetics Services.

The question remains regarding how their services may be improved (Table 3). At present, relatively small numbers of women are referred, partly because of the emphasis on family history for referral. Inclusion in FHCs of women at increased risk due to the presence of non-familial risk factors awaits the widespread introduction of MD and SNPs to risk prediction models. The introduction of new risk factors, such as MD and SNPs, is particularly important, as there is evidence that without them women are currently being given erroneous risk estimates that may result in imprecise treatment stratification. Clinical Genetics Services would be helped by more precise prediction of PVs. Consideration might be given to abandoning large panel tests and focussing on the nine genes in which the PVs are associated with a two-fold or more risk of BC [24].

We are currently testing the feasibility of introducing identification and referral of higher risk women as part of routine screening, i.e., research with a focus on implementation as part of routine care in a NHSBSP could bring about a “step change” if implemented [6]. There is already some evidence that communicating breast cancer risk estimates as part of routine screening does not produce the harms that have been anticipated [99]. For instance, communicating risk estimates in this setting did not produce adverse emotional effects or effects on screening uptake [100].

It appears that it is timely to consider introduction of the service into primary care as is seen in the USA [101]. A challenge to implementation is that the risk estimation and treatment algorithms have become more complex and efforts to introduce the two models we have focussed on here have led to legitimate difficulties amongst busy primary care physicians [102,103]. Even the mainstream estimation of cardiovascular risk on practice computer systems is apparently only applied to half of those in need and only half of these who need it are treated (Q-RISK; Hippisley-Cox 2017) [104] suggesting difficulties with the primary care approach.

A possible approach, pioneered in Melbourne, is to develop a simplified version of the Tyrer–Cuzick model, called iPrevent, and to make it widely available to all women. The model is user friendly and provides suggested treatment pathways in addition to an individual’s risk. If made widely available this could lead to patient-initiated referral for initial screening and SNP estimation to define definitive management [105]. Other measures may be to establish one-off breast density assessment for all women at a certain age (e.g., 40 years, as suggested above) to estimate BC risk and introduce further screening and preventive measures for those found to be at high risk [99,106].

## 4. Conclusions

We reported the activity in a clinic designed for referral of women concerned about their family history of breast cancer. The long period of the clinic illustrates the changes in risk estimation and management over the years. The time span also allows for multiple studies on the effectiveness of management, for example, the effectiveness of breast screening. It also allows for the study of and introduction of preventive approaches such as use of tamoxifen and anastrozole.

A major aim of the clinic is to reduce the incidence of and deaths from breast cancer. These will be reduced by screening, lifestyle change and chemoprevention. Improvements in their effectiveness depends upon more widespread introduction not only into the current at-risk population but also into the large proportion of women unknowingly at high risk

## Figures and Tables

**Table 1:** Number of referrals to the Family History Clinic (FHC) according to the source of referral, NICE guideline [3] risk category and *BCA1/2* status.

Group	Number	% of All Referrals	Known Gene in Family	%	High Risk	%	Moderate	%	Average	%	BRCA +v at Last Follow Up	%	Number
GP	8004	55.9%	165	2.1%	2646	33.1%	3542	44.3%	1651	20.6%	222	2.77%	8004
Surgery	2157	15.1%	38	1.8%	751	34.9%	831	38.6%	537	24.9%	55	2.55%	2157
Genetics	2868	20.0%	662	23.5%	1445	51.5%	594	21.2%	168	6.0%	406	14.16%	2868
PROCAS	448	3.1%	2	0.4%	311	69.4%	124	27.7%	11	2.5%	0	0.00%	448
Other	833	5.8%	54	6.5%	307	36.9%	299	35.9%	173	20.8%	53	6.36%	833
Totals	14,311		921	6.4%	5460	38.2%	5390	37.7%	2540	17.7%	736	5.14%	

GP, general practitioner; PROCAS, Prediction Risk Of Cancer At Screening.

**Table 2:** Number of women (A) randomised into the IBIS I or IBIS II trial 1992 and 2012; (B) number of women prescribed chemoprevention (CP) from 2013 either outside or as part of in-house trials.

A						B					
Group	Number Seen	Randomised to IBIS I/II Trial	Number Invited to Trial	% Joined Trial	Seen Since 2013	Prescribed Chemoprevention	%	Joined Chemoprevention Trial	%	Either Trial or CP	
Known gene in family	921	11	554	1.99%	364	2	0.55%	2	0.55%	4	1.10%
High	5488	465	3178	14.63%	2604	162	6.22%	168	6.45%	323	12.40%
Moderate	5370	431	3200	13.47%	2221	118	5.31%	113	5.09%	226	10.18%
Average	2532	112	1188	9.43%	609	0		0		0	
Total	14,311	1019	8120	12.55%	5798	282	4.86%	283	4.88%	553	9.54%

**Table 3:** Summary of “current practice” and issues to be addressed for each of the interventions discussed.

Intervention	Current Practice	Issues to Be Addressed
Referral	Referrals from primary & secondary care established	Only 20% of women with FH referred Very few with ‘other’ risk factors
Risk estimation	Evolved to include more risk factors eg. mammographic density & SNPs	Using all factors approximately 20% of population at moderate & high risk
Gene testing	BRCA1/2 & PALB2 available in NHS 10% threshold for PV used (NICE)	New data suggest panel of 9 genes be should be used
Screening	Annual mammography & MRI established	Breast density & SNPs being tested in trials of risk & density adapted screening
Lifestyle change	Observational studies suggest introduction would be valuable	Mechanisms for general application being tested
Chemoprevention	Longer term risk and benefits established	Application suboptimal—consider assessment at home and primary care
Risk reducing surgery	Offer at appropriate risk levels established	Continue improvements in psychological and surgical techniques
